# Adapting UK Clinical Practise in Chronic Myeloid Leukaemia in Chronic Phase to Contemporary Management Recommendations: The ADAPT 2.0 Survey

**DOI:** 10.1111/ejh.70226

**Published:** 2026-06-25

**Authors:** Dragana Milojkovic, Nicholas C. P. Cross, Ketul Desai, Kathryn Frewin, Mhairi Copland

**Affiliations:** ^1^ Centre for Haematology, Faculty of Medicine, Imperial College London, and Department of Haematology, Hammersmith Hospital, Imperial College Healthcare NHS Trust London UK; ^2^ Faculty of Medicine, University of Southampton Southampton UK; ^3^ Novartis Pharmaceuticals Ltd London UK; ^4^ Paul O'Gorman Leukaemia Research Centre, School of Cancer Sciences, College of Medical, Veterinary and Life Sciences, University of Glasgow Glasgow UK

**Keywords:** chronic myeloid leukaemia, patient management, survey, United Kingdom

## Abstract

**Objective:**

Chronic myeloid leukaemia in chronic phase (CML‐CP) treatment recommendations have changed substantially in recent years. The ADAPT 2.0 survey aimed to provide an updated understanding of CML‐CP management in the UK.

**Methods:**

This healthcare professional (HCP) survey, conducted between December 2023 and June 2024, assessed treatment goals, choice of tyrosine kinase inhibitors (TKIs), unmet needs and approaches to treatment‐free remission (TFR).

**Results:**

UK CML‐CP practise is generally aligned with the contemporary guidelines for CML management. Key treatment goals were major molecular response (54%–77% across treatment lines) and deep molecular response (5%–46%). The main factors influencing first‐line (1 L) TKI selection were treatment guidelines (97%), comorbidities (90%) and TFR as a treatment goal (82%). Therapy switch after one warning European LeukemiaNet (ELN) 2020 response was rare (10% vs. 56% in patients with > 1 warning response and 100% in failure response). *BCR::ABL1* mutation analysis was less common after one warning response versus > 1 warning response (54% vs. 82%). TKI toxicity, resistance, intolerance and T315I mutation management were key unmet needs in later treatment lines; achieving and maintaining TFR were key unmet needs in 1 L.

**Conclusion:**

The ADAPT 2.0 survey highlights potential areas of improvement in current CML‐CP management in the UK.

## 1 Introduction

Chronic myeloid leukaemia (CML) is a rare haematological malignancy, with a prevalence of 1.1–1.3/100 000 persons in the United Kingdom (UK) [1, 2]. The advent of tyrosine kinase inhibitors (TKIs) has dramatically improved overall survival in patients with CML, which now approaches that of the general population [3]. As a result of the substantial improvement in survival, treatment goals for CML have shifted to improving quality of life (QoL), with a particular focus on treatment‐free remission (TFR) [4].

The European LeukemiaNet (ELN) 2025 recommendations, the British Society for Haematology (BSH) 2020 guidelines and the ELN 2023 laboratory recommendations for CML highlight the best current practises for diagnosis, treatment and management of the disease, providing up‐to‐date guidance based on both expert consensus and a review of the latest clinical trial data [5, 6, 7]. The ELN 2025 recommendations are the latest available, and the ELN 2020 recommendations [8] were the most recent ones at the time this study was conducted. Changes from ELN 2013 [9] to ELN 2020 recommendations included a redefinition of treatment failure (*BCR::ABL1* international scale [IS] > 10% at 3 months instead of 6 months) to emphasise the importance of early intervention for patient outcomes. New TKIs were added to the list of available treatments and recommendations were made on which TKI to use for the management of patients with different *BCR::ABL1* mutations and for those with comorbidities and contraindications; a detailed diagnostic work‐up was also included to ensure comprehensive baseline assessments. Finally, TFR was introduced as a significant treatment goal in CML, with detailed recommendations for discontinuation criteria [8]. On the other hand, the ELN 2023 laboratory recommendations also included a diagnostic work‐up with details on pre‐treatment tests, guidance on disease monitoring and testing for *BCR::ABL1* kinase domain (KD) mutations and additional genomic abnormalities [6].

To understand the impact of the changing guidance on UK contemporary treatment practises in CML in chronic phase (CP), a practise‐reviewing survey of healthcare professionals (HCPs; ADAPT) was conducted in 2020 [10, 11]. The survey found that most respondents were aligned with the latest ELN recommendations, although several aspects of patient management remained suboptimal including the lack of well‐documented cardiovascular (CV) risk assessments, the infrequent use of chromosome banding analysis (CBA) in diagnostic work‐up, and the substantial proportion of clinicians not conducting *BCR::ABL1* mutational analysis in patients with ELN 2020 warning or failure response. Major molecular response (MMR, BCR::ABL1^IS^ ≤ 0.1%) was identified as the main treatment goal, and commonly reported challenges in patient management included TKI toxicity/intolerance and QoL.

The ADAPT 2.0 HCP survey aims to provide an updated understanding of current CML‐CP management and treatment pathways in the UK, including unmet medical needs, diagnosis and management of CML‐CP since the arrival of more recent treatment recommendations.

## 2 Methods

### 2.1 Survey

A structured survey was developed by Novartis UK (London, England) in accordance with Market Research Guidelines [12, 13, 14, 15]. A steering committee of physicians (composed of the three academic authors of this work) provided advice throughout survey development. The survey comprised 44 questions over seven sections, including the following topics: current background and practise with patients with CML‐CP, CML diagnostic testing, patient follow‐up and monitoring, treatment goals, choice of TKI therapy, unmet needs for patients with CML‐CP and approach to patients with intolerance or those who want to attempt TFR. The survey involved a combination of open‐ and closed‐ended questions, single‐ and multiple‐answer questions. Most questions required respondents to select answers from a pre‐defined list, with an option to provide further information if the ‘other’ field was chosen. The full survey questionnaire is presented in Supporting Information.

The 60‐min online survey was completed during structured appointments (video calls or face‐to‐face visits) with members of the Novartis medical scientific liaison (MSL) team between 6 December 2023 and 7 June 2024.

### 2.2 Participants

HCPs who were personally involved in the management of at least five patients with CML in the past 12 months were recruited by the MSL team. Participation was voluntary and no payments were made to encourage completion of the survey.

### 2.3 Analysis

Survey responses were analysed by third party company Adelphi Research. Responses were analysed descriptively; no statistical analyses were conducted. Categorical variables were described by frequencies and percentages, as appropriate. Content analysis and categorisation were used for free‐text responses.

### 2.4 Ethics

All participants provided informed consent. Records were anonymised, aggregated and analysed by research specialists at Adelphi Research. Institutional review board exemption was obtained before fieldwork commenced for all research materials used in the survey.

## 3 Results

A total of 39 HCPs responded to the survey, comprising 38 haematology consultants and 1 pharmacist/prescriber. Regarding primary practise setting, 49% of respondents worked in tertiary centres, 49% in district general hospitals and the remainder in other institution types. Approximately 40% of respondents had managed between 21 and 70 patients in the last 12 months preceding the survey. Overall, 18% of HCPs had seen more than 100 patients with CML‐CP in the clinic over the previous 12 months.

### 3.1 Diagnostic Testing

**FIGURE 1:**
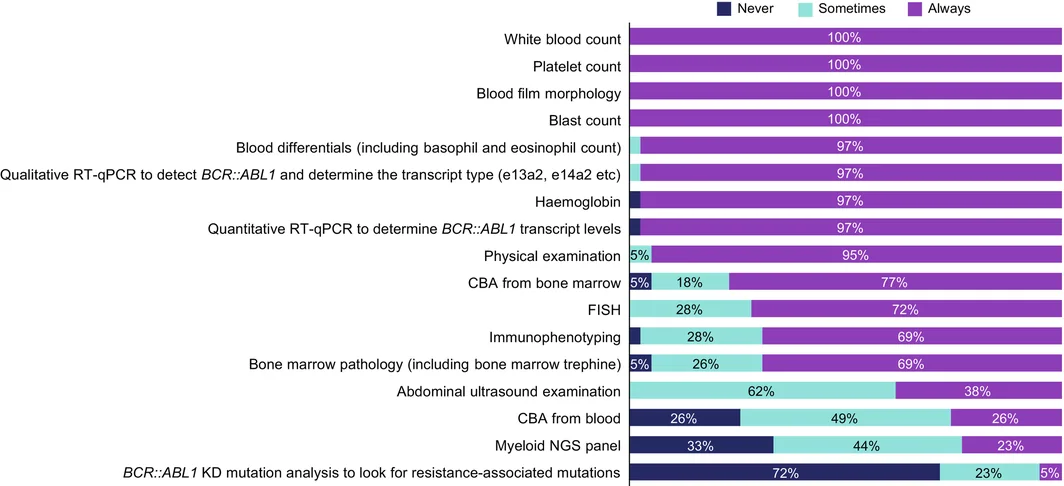
Use of diagnostic tests in CML‐CP work‐up. Figure shows percentage of responding HCPs; more than one answer could be selected. CBA, chromosome banding analysis; CML, chronic myeloid leukaemia; CP, chronic phase; FISH, fluorescence in situ hybridisation; HCP, healthcare professional; NGS, next‐generation sequencing; RT‐qPCR, real‐time quantitative polymerase chain reaction.

All responding HCPs reported routinely performing white blood cell (WBC) and platelet counts, as well as blood film morphology including blast count, as part of the diagnostic work‐up for CML (Figure 1). Conventional cytogenetic analysis (CBA) was also consistently performed, either from blood or bone marrow. In contrast, abdominal ultrasound, myeloid next‐generation sequencing (NGS) panel and *BCR::ABL1* KD mutational analysis were not routinely performed.

Whilst CV history was consistently recorded by all HCPs in patient notes, only 82% captured family CV history (Figure S1). Other important indicators such as electrocardiogram (ECG), body mass index, lipid profiles and physical activity were less commonly recorded in patient notes. With regard to CV risk assessment, 54% of HCPs reported using the QRISK3 tool (Figure S2). Most of the HCPs who do not use any CV risk assessment tool (33%) either conduct an initial baseline assessment and subsequently refer the patient to a general practitioner (GP) or delegate both assessment and risk management entirely to the GP. Notably, 23% of these HCPs believe that the use of a formal tool would not significantly influence their clinical decisions.

Approximately 85% of HCPs reported using the EUTOS long‐term survival (ELTS) score [16] as a risk assessment tool, alone or combined with others, whilst approximately 62% use the Sokal score (Figure S3); a small minority (5%) uses the Hasford score, always combined with ELTS and Sokal.

### 3.2 Key Treatment Goals

**FIGURE 2:**
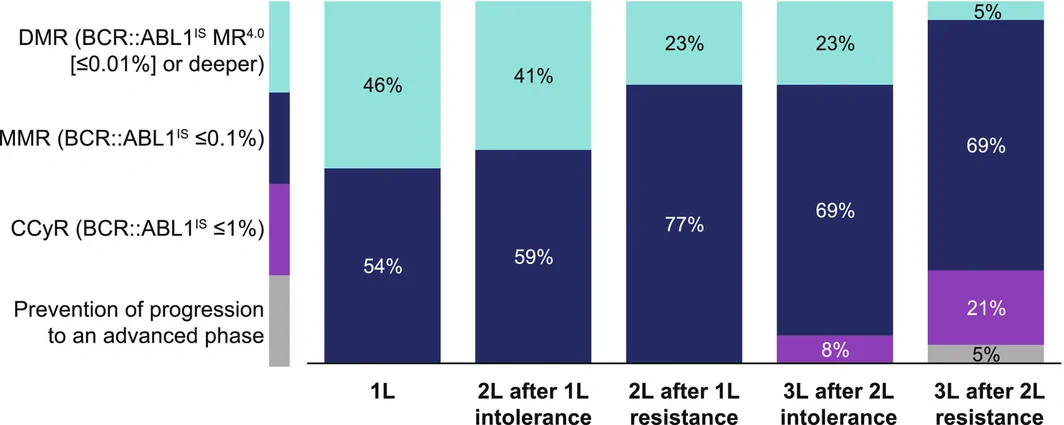
Treatment response goals for patients with CML‐CP. Figure shows percentage of responding HCPs. 1 L, first line; 2 L, second line; 3 L, third line; CCyR, complete cytogenetic response; CML‐CP, chronic myeloid leukaemia in chronic phase; DMR, deep molecular response; HCP, healthcare professional; IS, international scale; MMR, major molecular response.

MMR was the primary treatment goal across all lines of therapy, cited by 54%–77% of respondents depending on the treatment line and peaking for patients in second line (2 L) following first‐line (1 L) resistance (Figure 2). Deep molecular response (DMR; BCR::ABL1^IS^ ≤ 0.01%) was also a key goal, particularly in earlier lines, though its prioritisation declined from 46% at 1 L to 5% at third line (3 L) after 2 L resistance. Complete cytogenetic response (CCyR; BCR::ABL1^IS^ ≤ 1%) emerged as a treatment goal for a minority of HCPs at 3 L, especially after 2 L resistance.

### 3.3 Factors Affecting TKI Selection for Patients With CML‐CP

The primary factors influencing 1 L TKI selection were treatment guidelines (97%), comorbidities (90%) and TFR as a treatment goal (82%) (Table S1). Tolerability, safety profile and CV risk score became more important factors affecting TKI selection after 1 L intolerance or resistance. Second‐generation (2G) TKIs were more likely to be selected in 1 L for patients planning pregnancy or those with high/intermediate‐risk Sokal or ELTS scores, whilst opinions varied regarding the use of 2G TKIs in patients aiming for TFR and in younger patients; responses were similar regardless of the presence of CV risk factors (Figure S4). Tolerability was also a more prominent factor affecting TKI selection after intolerance to prior therapy. In contrast, CML risk progression scores were more influential at 1 L than in later lines, even after resistance.

### 3.4 Patient Follow‐Up and Monitoring

**FIGURE 3:**
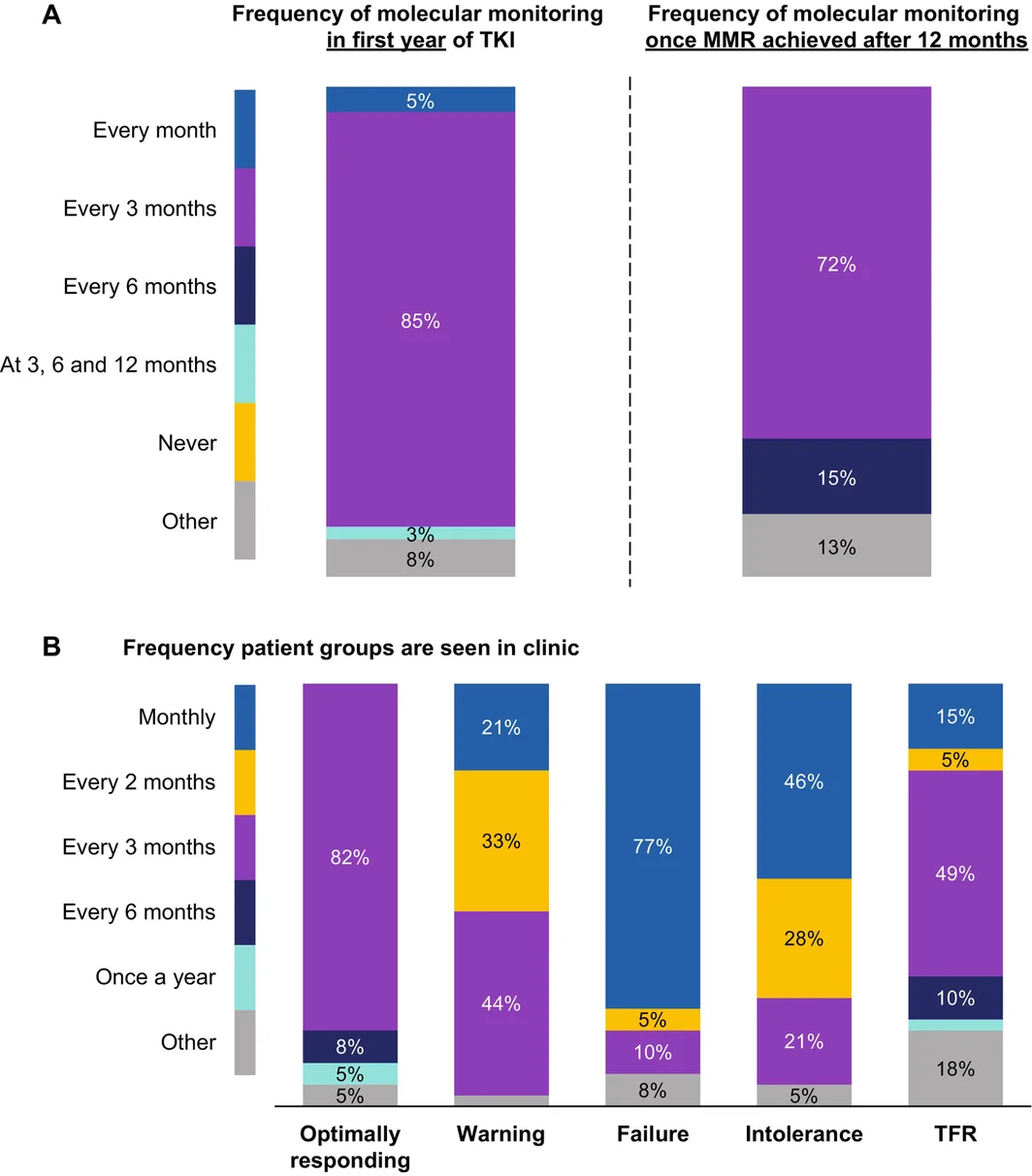
Patient monitoring. (A) Frequency of patient monitoring; (B) frequency patient groups are seen in the clinic. Figure shows percentage of responding HCPs. CML‐CP, chronic myeloid leukaemia in chronic phase; HCP, healthcare professional; MMR, major molecular response; TFR, treatment‐free remission; TKI, tyrosine kinase inhibitor.

Most respondents reported performing *BCR::ABL1* monitoring every 3 months during the first year of TKI treatment and continuing at this frequency after patients achieved MMR at 12 months (85% and 72%, respectively; Figure 3A). Approximately 15% of respondents transitioned to 6‐monthly monitoring after MMR was achieved at 12 months. Tests other than real‐time quantitative polymerase chain reaction (RT‐qPCR), such as myeloid NGS panel or bone marrow pathology, were not routinely used for follow‐up by most respondents (Figure S5).

With regard to monitoring frequency by response type, patients with CML‐CP showing an optimal response per ELN 2020 criteria were typically reviewed every 3 months (82%; Figure 3B); however, only 13% were seen in person (Figure S6). Patients with failure response or intolerance were reviewed more frequently (every 1 or 2 months by 82% and 74% of respondents, respectively) and mostly in person (79% and 67%, respectively). Monitoring approaches varied for patients with a warning response: 44% were seen every 3 months, whilst over half were reviewed every 1 or 2 months. Patients attempting TFR were most commonly monitored every 3 months; it should be noted that the question did not specify the time frame for patients attempting TFR, so the response likely represents an average.

Almost half (49%) of HCPs reported not having access to or not using additional tools and/or support staff for CML monitoring (Figure S7); amongst those who use one of these additional resources, CML clinical nurse specialist (CNS) assessment was the most frequently selected.

### 3.5 Patient Management

**FIGURE 4:**
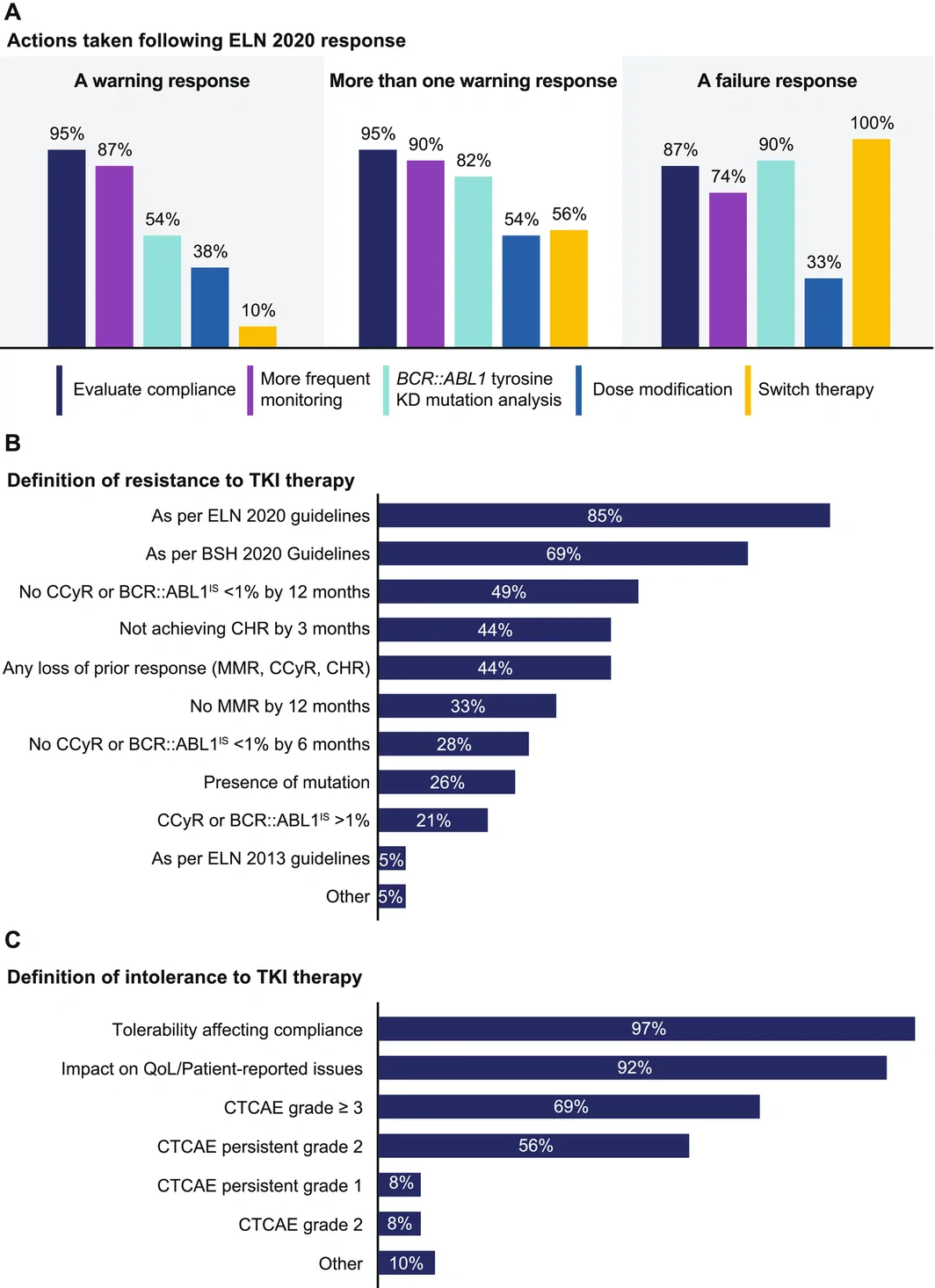
Patient management according to response. (A) Actions taken following ELN 2020 response; (B) definition of resistance to TKI therapy; (C) definition of intolerance to TKI therapy. For each response on definitions, more than one answer could be selected. Figure shows percentage of responding HCPs. BSH, British Society for Haematology; CHR, complete haematologic response; CCyR, complete cytogenetic response; CTCAE, Common Terminology Criteria for Adverse Events; ELN, European LeukemiaNet; HCP, healthcare professional; IS, international scale; MMR, major molecular response; QoL, quality of life; TKI, tyrosine kinase inhibitor.

*BCR::ABL1* KD mutational analysis was increasingly likely in patients exhibiting more than one ELN 2020‐defined warning response or failure response compared to those with one warning response (Figure 4A). Most HCPs (90%) reported waiting or sometimes waiting for mutational analysis results before switching TKI (Figure S8). Notably, dose modifications were a less likely approach following a failure ELN response, with patients more commonly switched to another therapy. Only 54% of HCPs conduct *BCR::ABL1* mutational analysis after a single warning response. When asked for reasons for not performing *BCR::ABL1* mutational analysis in TKI‐resistant patients, almost all responding HCPs (38/39, 97%) stated that this analysis was not applicable (Figure S8).

The majority of respondents defined resistance to TKI therapy as per the ELN 2020 recommendations (85%) or BSH 2020 guidelines (69%; Figure 4B). Around half (44%–49%) defined resistance as either no CCyR or no BCR::ABL1^IS^ < 1% by 12 months, not achieving complete haematologic response (CHR) by 3 months, or any loss of prior response. One third of respondents defined resistance as no MMR by 12 months, whereas 26% defined it as the presence of a mutation. On the other hand, almost all respondents defined intolerance to TKI therapy primarily in terms of tolerability affecting compliance and impacting QoL or as patient‐reported issues (Figure 4C). Higher‐grade or persistent grade 2 adverse events (AEs) were also commonly cited as defining intolerance, whereas non‐persistent or lower‐grade AEs were generally not considered indicative of intolerance.

Switching TKI therapy was the most common approach to managing intolerance (92% across therapy lines), followed closely by dose reduction (90%–92%) and consideration of drug interactions (90%) (Figure S9). For patients experiencing Grade ≥ 3 AEs, TKI switching was the predominant approach (97%). Notably, the proportion of respondents who switched TKI increased from 26% in patients with Grade 1 or 2 AEs to 62% in those with persistent Grade 1 or 2 AEs.

### 3.6 Treatment‐Free Remission

**FIGURE 5:**
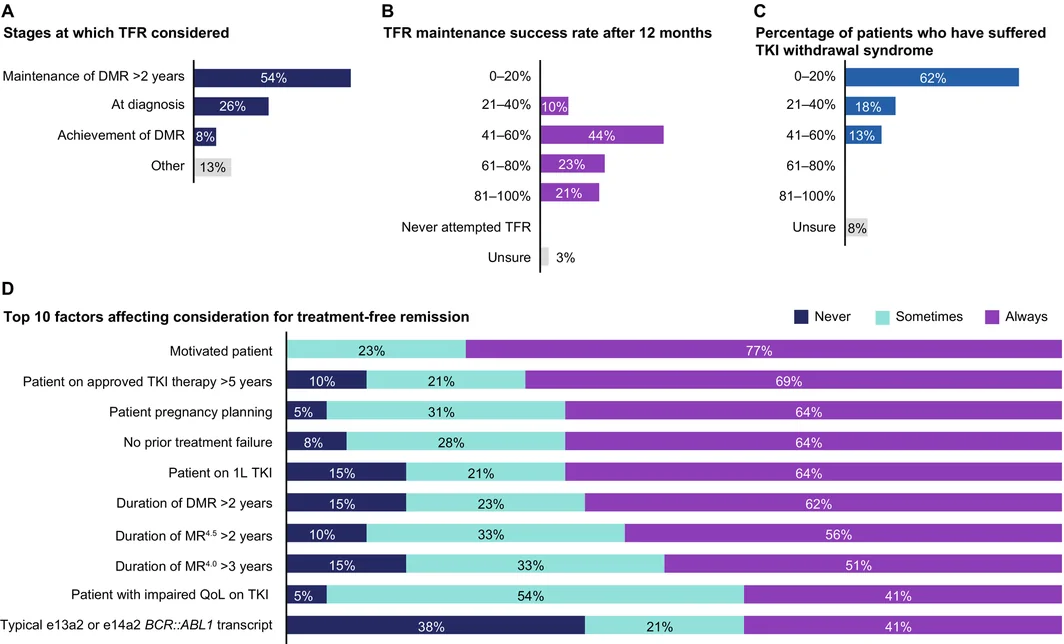
Treatment‐free remission. (A) Stages at which TFR was considered; (B) TFR maintenance success rate after 12 months; (C) percentage of patients who have suffered TKI withdrawal syndrome; (D) factors affecting consideration for TFR. Figure shows percentage of responding HCPs. 1 L, first line; DMR, deep molecular response; HCP, healthcare professional; MR, molecular response; QoL, quality of life; TFR, treatment‐free remission; TKI, tyrosine kinase inhibitor.

The most common guidelines followed for TFR were the BSH guidelines (59%), the DESTINY protocol [17] (56%) and the ELN 2020 recommendations (54%). Over half of responding HCPs considered TFR after maintaining DMR for > 2 years (Figure 5A). However, TFR was not routinely considered at diagnosis, with only 10/39 HCPs (26%) stating they consider TFR for a patient at this time. Reported TFR maintenance success rates after 12 months typically exceeded 40%, with over 40% of HCPs reporting success rates between 61% and 100% (Figure 5B). Most HCPs (64%) agreed that few patients (0% to 20%) experience TKI withdrawal syndrome (Figure 5C) [18].

Patient motivation was the most widely reported factor when considering TFR, followed by an overall treatment duration above 5 years (Figure 5D). Patient age, presence of *BCR::ABL1* KD mutations, high‐risk features, or ELTS score were less important factors when considering TFR (Figure S10).

### 3.7 Unmet Needs

**FIGURE 6:**
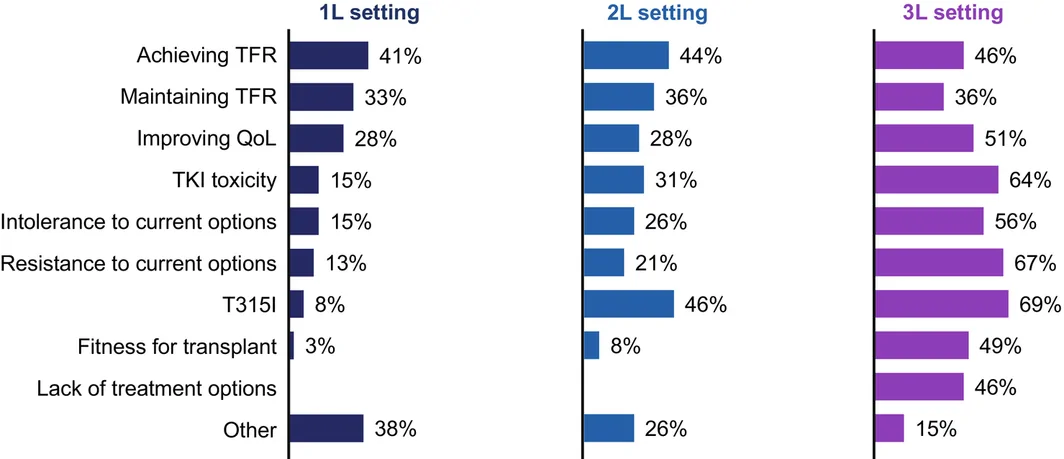
Unmet need in patients with CML. Figure shows percentage of responding HCPs. 1 L, first line; 2 L, second line; 3 L, third line; CML, chronic myeloid leukaemia; HCP, healthcare professional; QoL, quality of life; TFR, treatment‐free remission; TKI, tyrosine kinase inhibitor.

Unmet needs varied across treatment lines. In 1 L and 2 L, achieving and maintaining TFR were rated as high unmet needs; although these priorities persisted into 3 L, their relative importance decreased (Figure 6). The T315I mutation was a top unmet need in 2 L and 3 L (46% and 69%, respectively). Resistance and intolerance to available therapies emerged as key needs in 3 L, together with TKI toxicity and patient fitness for transplant; lack of treatment options was a concern in 3 L only. In terms of educational requirements of clinical teams managing CML, CV risk management and comorbidity management were the top unmet needs (Figure S11). It was noted that CNSs in particular would benefit from further education.

## 4 Discussion

Since the publication of the ELN recommendations in 2013 [9], there have been several advances in the management of patients with CML‐CP in the UK. The findings of the ADAPT 2.0 survey suggest that management of CML in the UK is broadly in line with ELN 2020 and 2023 recommendations, indicating a high level of awareness amongst HCPs. The survey respondents came from a diverse mix of teaching and district general hospitals, which provided a comprehensive understanding of CML management across different settings. This is important given the potential differences in CML care between highly specialised tertiary centres and broader, community‐based district general hospitals [19].

Both the ELN 2020 and 2023 laboratory recommendations mention a detailed diagnostic work‐up for CML. Despite recent recommendations for patients to undergo formal CV risk assessment before starting treatment with second or third‐generation TKIs, almost one‐third of HCPs do not use a validated CV risk assessment tool; however, the use of the QRISK3 tool increased from 32% in the 2020 ADAPT survey to 54% [10, 11]. The use of the ELTS score also increased since the ADAPT survey (alone or in combination, from 35% to 85%), but most HCPs still use the Sokal scale to assess risk scoring, despite the ELN 2020 recommendations' preference for the ELTS score [8].

Whilst MMR remains the top treatment goal across therapy lines, a higher number of HCPs now aim for DMR in 1 L and 2 L after 1 L intolerance as a key treatment goal compared with the original ADAPT survey (46% and 41% in 1 L and 2 L after 1 L intolerance vs. 37% and 35% in ADAPT, respectively) [10, 11]. Similar proportions of physicians reported MMR as a top treatment goal in the global CML Survey on Unmet Needs (SUN); however, more HCPs in CML SUN reported considering DMR as a key treatment goal for patients in 2 L and 3 L (54% and 52%, respectively) [20].

When selecting a TKI, there is increased consideration of treatment guidelines, tolerability and patient choice across therapy lines, as well as of patient comorbidities, CV risk and TKI safety profile in later lines compared with the ADAPT survey [10, 11]. A substantial percentage of HCPs also consider TFR as a key treatment goal, in line with ELN 2020 recommendations [8], and consider it as one of the key factors in TKI selection, particularly for 1 L patients. Of note, the latest version of the ELN recommendations collates recent evidence and highlights that the ideal candidates for attempting TFR are patients who achieved early response (BCR::ABL1^IS^ < 1% by 12 months) [7].

Overall, monitoring frequency was linked to actions taken with different ELN 2020 responses as per treatment recommendations [8]. However, 15% of HCPs monitor patients in MMR every 6 months instead of the 3 months recommended by ELN 2020. Interestingly, the ELN 2025 recommendations [7] show a more flexible, individualised approach to monitoring patients who have achieved MMR than the 2020 version, mentioning that monitoring every 4 to 6 months is justified in patients with stable response. Increased frequency of monitoring for patients in ELN 2020 warning or failure response was reported more often than in the original 2020 ADAPT survey (1 warning response: 87% vs. 65%; > 1 warning response: 90% vs. 46%; failure response: 74% vs. 41%, respectively) [10, 11]. Increased proportions of HCPs also reported evaluating compliance in these patients compared to the ADAPT survey. Despite overall alignment with ELN 2020 and 2023 recommendations, not all respondents conduct *BCR::ABL1* KD mutation testing in patients with warning/failure ELN 2020 response as recommended; similar results were reported in the ADAPT survey [10, 11]. It should be noted that the ELN 2025 recommendations now use different language for responses, replacing ‘optimal’, ‘warning’ and ‘failure’ with ‘favourable’, ‘warning’ and ‘unfavourable’ [7]. This reflects both the most recent evidence and the increased focus on personalised patient management.

TKI switch was a relatively common approach in patients with more than one ELN 2020 warning response, although it was less frequently reported than in the original ADAPT survey (56% vs. 72%, respectively) [10, 11]. This may reflect a more conservative approach to treatment switching, likely to avoid the higher failure rates associated with increasing treatment lines [21, 22]. It may also reflect an ageing patient population, where clinicians would try to avoid switching from imatinib to 2G TKIs due to the associated toxicity in patients with higher CV risk and other comorbidities. Of note, ELN 2025 recommendations support a more individualised approach to treatment switch, based on all previous monitoring results rather than just the latest ones [7].

Clear guidance exists regarding the definitions of resistance for patients with CML, and this guidance is followed by most responding HCPs; however, no such guidance exists in terms of defining intolerance. Most HCPs identify intolerance as affecting adherence and impacting QoL, as well as being associated with higher‐grade AEs. However, non‐persistent or lower‐grade AEs were unlikely to be interpreted as intolerance to TKI therapy despite their impact on patients' daily lives [23, 24, 25, 26]. Consistent with these results, a relatively low proportion of HCPs would consider a therapy switch in patients experiencing Grade 1/2 events, even persistent ones. The CML SUN survey identified side effects from treatment as one of the top three reasons for patient non‐compliance; importantly, only 40% of patients in 1 L and 24% of patients in 2 L reported that their physician showed empathy and tried to understand the impact of treatment side effects on their lives [20]. As well as improved understanding of the impact of treatment‐related AEs on patients' daily lives, a new instrument or framework to assess tolerability in patients with CML is needed to provide a more structured definition of intolerance and guidance to clinicians for optimal patient management.

TKI toxicity was no longer a top unmet need for patients in 1 L and 2 L as had been reported in the original ADAPT survey [10, 11]; this suggests that novel TKIs and improved AE management have at least partially overcome this challenge. As expected given the shift in treatment goals, TFR is now a top unmet need across all therapy lines. Although reported TFR success rates were generally high, some HCPs likely described limited attempts at TFR with highly selective patient criteria, which may contribute to their perception of higher success rates.

One potential limitation of this study is the relatively low number of responding HCPs, which may impact the generalisability of the results; however, the number was similar to that of the ADAPT respondents (*n* = 46). A significant proportion of respondents had expertise in CML management, treating a relatively large number of patients in each year and/or working in a tertiary centre setting; responses may thus not be generalisable to the general UK physician population. Decisions on patient management hinge on multiple factors (related to patients, such as demographics and comorbidities, and to care settings, such as availability of resources) and their full complexity may not have been captured in this survey. It should be noted that practise was documented through self‐report rather than direct observation. Although the survey was designed to standardise responses and enable comparison across participants, some questions may have been open to differing interpretation, and the use of pre‐defined answer options may have limited the ability of respondents to fully capture the complexity of their routine clinical practise. As with all questionnaire‐based studies, responses may have been subject to recall bias, reporting bias and differences between reported and actual practise. Finally, potential bias from the Novartis MSLs conducting the interviews cannot be discarded.

Many clinicians in the UK consider CML to be less complex and severe, and to have an overall lesser impact on patients' lives compared to other blood malignancies; [2] however, management of CML still presents challenges, particularly in terms of treatment resistance and impact on patient QoL. Overall, the results from the ADAPT 2.0 survey provide insights into the current management of patients with CML, showing evolving changes in practise that follow updated recommendations and highlighting areas of potential improvement, such as increasing the use of validated CV risk assessment tools, broader adoption of the ELTS score for risk stratification, and more consistent application of comprehensive diagnostic and monitoring practises in line with guidelines and recommendations.

## Funding

This study was funded by Novartis Pharmaceuticals UK Ltd.

## Conflicts of Interest

Dragana Milojkovic: Honoraria (Pfizer, Ascentage, Novartis and Incyte). Nicholas C. P. Cross: Research funding and honoraria (Novartis); honoraria (Ascentage). Ketul Desai and Kathryn Frewin: Employee of Novartis. Mhairi Copland: Research funding (Cyclacel and Incyte); advisory board member (Ascentage, Novartis, Incyte, Jazz Pharmaceuticals, Pfizer and Servier); honoraria (Astellas, Novartis, Incyte, Pfizer, Janssen and Jazz Pharmaceuticals).

## Supporting information


**Figure S1:** Information routinely captured in patient's notes regarding CV risk.
**Figure S2:** CV assessment tools used to assess patients with CML prior to first TKI.
**Figure S3:** Risk scoring tools used to assess patients with CML‐CP prior to first TKI.
**Figure S4:** Frequency of considering 2G TKI as 1 L treatment for CML‐CP.
**Figure S5:** Additional tests performed for routine follow‐up.
**Figure S6:** Ways to review patients in the clinic according to interface use and pathways to obtain blood samples.
**Figure S7:** Tools and resources used to monitor patients with CML and additional tools required by HCPs.
**Figure S8:** Reasons for not performing *BCR::ABL1* tyrosine kinase domain mutational analysis in TKI‐resistant patients.
**Figure S9:** Actions considered if patient is intolerant to TKI therapy by therapy line and AE severity.
**Figure S10:** Less important factors affecting consideration for TFR.
**Figure S11:** Educational requirements of clinical teams managing CML and HCPs who would benefit from further education.
**Table S1:** Factors affecting TKI selection for patients with CML‐CP.

**Data S1:** Checklist for reporting of survey studies (CROSS).

## Data Availability

Data collection was undertaken by Adelphi Research as part of a survey conducted in collaboration with Novartis Pharmaceuticals UK Ltd, entitled ADAPT 2.0. All data supporting the findings of this study are the intellectual property of Novartis Pharmaceuticals UK. All requests for access should be addressed directly to Ketul Desai.
