# Supplementary material for: Adapting UK Clinical Practise in Chronic Myeloid Leukaemia in Chronic Phase to Contemporary Management Recommendations: The ADAPT 2.0 Survey
Source: Eur J Haematol. 2026 Jun 25;117(4):1072–80. doi: 10.1111/ejh.70226 (PMC13542934; doi:10.1111/ejh.70226)
Supplement: Supplementary file 1 [file EJH-117-1072-s001.docx]

**Section 1: General Background**

**A1**

How would you categorise your hospital?

*Please select one.*

| **1** | District General |
| --- | --- |
| **2** | Tertiary Centre |
| **95** | Other |

**A2**

How many newly diagnosed CML-CP patients do you see per year?

*Please select one.*

| **1** | ≤ 5 |
| --- | --- |
| **2** | 5-10 |
| **3** | 11-20 |
| **4** | 21-50 |
| **5** | > 50 |

**A3**

How many CML-CP patients currently attend your clinic per year?

*Please select one.*

| **1** | ≤ 50 |
| --- | --- |
| **2** | 51-100 |
| **3** | > 100 |

**Section 2: Diagnostic Testing**

**B1**

How often do you use the following tests in your CML diagnostic work up?

|  |  | | **Never** | | **Sometimes** | **Always** | |
| --- | --- | --- | --- | --- | --- | --- | --- |
| **1** | Physical examination | 🞏 | | 🞏 | | 🞏 |  |
| **2** | Abdominal ultrasound examination | 🞏 | | 🞏 | | 🞏 |  |
| **3** | White blood count | 🞏 | | 🞏 | | 🞏 |  |
| **4** | Platelet count | 🞏 | | 🞏 | | 🞏 |  |
| **5** | Haemoglobin | 🞏 | | 🞏 | | 🞏 |  |
| **6** | Blood differentials (inc. basophil and eosinophil count) | 🞏 | | 🞏 | | 🞏 |  |
| **7** | Blood film morphology | 🞏 | | 🞏 | | 🞏 |  |
| **8** | Bone marrow pathology (inc. bone marrow trephine) | 🞏 | | 🞏 | | 🞏 |  |
| **9** | Blast count | 🞏 | | 🞏 | | 🞏 |  |
| **10** | Immunophenotyping | 🞏 | | 🞏 | | 🞏 |  |
| **11** | Cytogenetics: Chromosome-banding analysis (CBA) from Blood | 🞏 | | 🞏 | | 🞏 |  |
| **12** | Cytogenetics: Chromosome-banding analysis (CBA) from Bone Marrow | 🞏 | | 🞏 | | 🞏 |  |
| **13** | Fluorescence in situ hybridization (FISH) | 🞏 | | 🞏 | | 🞏 |  |
| **14** | Molecular testing: Qualitative RT-qPCR to detect *BCR::ABL1* and determine the transcript type (e13a2, e14a2 etc) | 🞏 | | 🞏 | | 🞏 |  |
| **15** | Molecular testing: Quantitative RT-qPCR to determine *BCR::ABL1* transcript levels | 🞏 | | 🞏 | | 🞏 |  |
| **16** | Molecular testing: *BCR::ABL1* tyrosine kinase domain mutation analysis to look for resistance-associated mutations | 🞏 | | 🞏 | | 🞏 |  |
| **17** | Myeloid Next-Generation Sequencing (NGS) Panel | 🞏 | | 🞏 | | 🞏 |  |
| **95** | Other molecular tests | 🞏 | | 🞏 | | 🞏 |  |

**B2**

Prior to prescribing the first TKI, what information would routinely be captured in the patient’s notes regarding cardiovascular (CV) risk?

*Select all that apply*

| **1** | Blood pressure |
| --- | --- |
| **2** | Body mass index (BMI) |
| **3** | Personal CV history |
| **4** | Family CV history |
| **5** | Comorbidities (inc. diabetes) |
| **6** | HBA1c Level |
| **7** | Total cholesterol |
| **8** | LDL cholesterol |
| **9** | Smoking status |
| **10** | Physical activity levels |
| **11** | Electrocardiogram (ECG) |
| **12** | Echocardiogram (ECHO) |
| **13** | Concomitant medications |
| **14** | Alcohol history |
| **96** | None of the above |

**B3**

Do you use any of the following validated CV assessment tools to assess patients with CML prior to first TKI?

*Select all that apply.*

| **1** | Qrisk3 |
| --- | --- |
| **2** | ASSIGN |
| **3** | No CV assessment tool used |
| **95** | Other (please specify) |

**B4**

What is your rational for not using any CV risk assessment tools?

*Select all that apply.*

| **1** | Rely on clinical experience |
| --- | --- |
| **2** | Does not impact treatment decision |
| **3** | Lack of Time |
| **4** | Lack of Awareness |
| **5** | Unsure of applicability |
| **6** | Perform baseline CV risk parameters and refer to GP |
| **7** | Refer to GP to perform and manage CV risk |
| **95** | Other |

**B5**

Do you use any of the following risk scoring tools for CML-CP patients?

*Select all that apply*

| **1** | Sokal |
| --- | --- |
| **2** | EUTOS Long-Term Survival (ELTS) |
| **95** | Other (please specify) |
| **96** | No risk assessment |

**B6**

What is your rational for not using any risk scoring tools?

*Select all that apply.*

| **1** | Rely on clinical experience |
| --- | --- |
| **2** | Does not impact treatment decision |
| **3** | Lack of Time |
| **4** | Lack of Awareness |
| **5** | Unsure of applicability |
| **95** | Other |

**Section 3: Treatment Goals**

|  | **C1**  What is your **main** treatment response goal for CML-CP patients?  *Please select one per line of treatment.* | | | | | | | | |  |
| --- | --- | --- | --- | --- | --- | --- | --- | --- | --- | --- |
|  |  | 1^st^ Line | 2^nd^ Line | | | | 3^rd^ Line | | |  |
|  |  |  | after **1L** **intolerance** | | after **1L** **resistance** | | after **2L** **intolerance** | after **2L** **resistance** | |  |
| **1** | Complete cytogenetic response (CCyR) (BCR-ABL1 IS ≤1%) | 🞏 | | 🞏 | | 🞏 | 🞏 | | 🞏 |  |
| **2** | Major molecular response (MMR) (BCR-ABL1 IS ≤0.1%) | 🞏 | | 🞏 | | 🞏 | 🞏 | | 🞏 |  |
| **3** | DMR (BCR-ABL1 IS MR4.0 (≤0.01%) or deeper) | 🞏 | | 🞏 | | 🞏 | 🞏 | | 🞏 |  |
| **4** | Prevention of progression to an advanced phase | 🞏 | | 🞏 | | 🞏 | 🞏 | | 🞏 |  |
|  | **C2**  Which other factors play a part in your decision-making process when selecting a TKI for a CML-CP patient?  *Select all that apply per line of treatment* | | | | | | | | |  |
|  |  | 1^st^ Line | | 2^nd^ Line | | | 3^rd^ Line | | |  |
|  |  |  |  | after **1L** **intolerance** | | after **1L** **resistance** | after **2L** **intolerance** | | after **2L** **resistance** |  |
| **1** | Efficacy | 🞏 | | 🞏 | | 🞏 | 🞏 | | 🞏 |  |
| **2** | Safety profile | 🞏 | | 🞏 | | 🞏 | 🞏 | | 🞏 |  |
| **3** | Tolerability | 🞏 | | 🞏 | | 🞏 | 🞏 | | 🞏 |  |
| **4** | QOL | 🞏 | | 🞏 | | 🞏 | 🞏 | | 🞏 |  |
| **5** | Dosing regimen | 🞏 | | 🞏 | | 🞏 | 🞏 | | 🞏 |  |
| **6** | Comorbidities | 🞏 | | 🞏 | | 🞏 | 🞏 | | 🞏 |  |
| **7** | Age | 🞏 | | 🞏 | | 🞏 | 🞏 | | 🞏 |  |
| **8** | CML progression risk score | 🞏 | | 🞏 | | 🞏 | 🞏 | | 🞏 |  |
| **9** | CV risk score | 🞏 | | 🞏 | | 🞏 | 🞏 | | 🞏 |  |
| **10** | Patient choice | 🞏 | | 🞏 | | 🞏 | 🞏 | | 🞏 |  |
| **11** | Cost | 🞏 | | 🞏 | | 🞏 | 🞏 | | 🞏 |  |
| **12** | Guidelines | 🞏 | | 🞏 | | 🞏 | 🞏 | | 🞏 |  |
| **13** | Aiming for TFR | 🞏 | | 🞏 | | 🞏 | 🞏 | | 🞏 |  |
| **14** | Standard local management choice | 🞏 | | 🞏 | | 🞏 | 🞏 | | 🞏 |  |
| **15** | Physician experience | 🞏 | | 🞏 | | 🞏 | 🞏 | | 🞏 |  |
| **95** | Other | 🞏 | | 🞏 | | 🞏 | 🞏 | | 🞏 |  |

**C3**

For each patient characteristic, how often would you consider for a 2G-TKI in 1st line treatment for CML-CP?

|  |  | | **Never** | | **Sometimes** | **Always** | |  | |
| --- | --- | --- | --- | --- | --- | --- | --- | --- | --- |
| **1** | High/Int Sokal or ELTS score | 🞏 | | 🞏 | | 🞏 | Single select per row  Show to all Respondents  Code 4 and 5 only force answer in ‘other specify’ if ‘Sometimes’ or ‘Always’ selected  Code 4 and 5 ‘please specify age’ only allow numeric answer  Allow alphanumeric input for codes 4 and 5 | |  |
| **2** | Low Sokal or ELTS score | 🞏 | | 🞏 | | 🞏 |  |  |  |
| **3** | Patient pregnancy planning | 🞏 | | 🞏 | | 🞏 |  |  |  |
| **4** | ‘Younger’ Patients (please specify age) | 🞏 | | 🞏 | | 🞏 |  |  |  |
| **5** | ‘Older patients’ (please specify age) | 🞏 | | 🞏 | | 🞏 |  |  |  |
| **6** | Patients without CV risk factors | 🞏 | | 🞏 | | 🞏 |  |  |  |
| **7** | Patients with CV risk factors | 🞏 | | 🞏 | | 🞏 |  |  |  |
| **8** | Higher risk chronic phase | 🞏 | | 🞏 | | 🞏 |  |  |  |
| **9** | Patients who would like to attempt TFR | 🞏 | | 🞏 | | 🞏 |  |  |  |
| **95** | Other | 🞏 | | 🞏 | | 🞏 |  |  |  |

**Section 4: Patient Follow Up and Monitoring**

**D1**

At what time points do you perform molecular monitoring of a CML-CP patient in their first year on a TKI (any line)?

*Please select one.*

| **1** | Every month |
| --- | --- |
| **2** | Every 3 months |
| **3** | Every 6 months |
| **4** | At 3, 6 and 12 months |
| **96** | Never |
| **95** | Other |

**D2**

Once patients have achieved MMR after 12 months (and long-term follow up) how frequently would they be subsequently monitored by molecular assay (*BCR::ABL1* IS by RQ-PCR)?

*Please select one.*

| **1** | Every 3 months |
| --- | --- |
| **2** | Every 6 months |
| **3** | Every 12 months |
| **96** | Never |
| **95** | Other |

**D3**

Do you perform any additional tests other than molecular monitoring for routine follow up?

|  |  | | **Never** | | **Sometimes** | **Always** | |
| --- | --- | --- | --- | --- | --- | --- | --- |
| **1** | Blood film morphology | 🞏 | | 🞏 | | 🞏 |  |
| **2** | Bone marrow pathology (inc. bone marrow trephine) | 🞏 | | 🞏 | | 🞏 |  |
| **3** | Cytogenetics: Chromosome-banding analysis (CBA) from Blood | 🞏 | | 🞏 | | 🞏 |  |
| **4** | Cytogenetics: Chromosome-banding analysis (CBA) from Bone Marrow | 🞏 | | 🞏 | | 🞏 |  |
| **5** | Fluorescence in situ hybridization (FISH) | 🞏 | | 🞏 | | 🞏 |  |
| **6** | Myeloid Next-Generation Sequencing (NGS) Panel | 🞏 | | 🞏 | | 🞏 |  |
| **95** | Other tests (please specify) | 🞏 | | 🞏 | | 🞏 |  |

**D4**

Which clinics do you usually manage and review the following patient groups in?

*Please select all that apply per patient group.*

|  |  | **Optimally responding CP-CML** | **Warning CML patient** | **Failure CP-CML** | **Intolerant patient** | **TFR patient** |
| --- | --- | --- | --- | --- | --- | --- |
| **1** | General Haematology Clinic | 🞏 | 🞏 | 🞏 | 🞏 | 🞏 |
| **2** | Myeloid Clinic | 🞏 | 🞏 | 🞏 | 🞏 | 🞏 |
| **3** | Myeloproliferative neoplasms (MPN) clinic | 🞏 | 🞏 | 🞏 | 🞏 | 🞏 |
| **4** | Dedicated CML clinic | 🞏 | 🞏 | 🞏 | 🞏 | 🞏 |
| **5** | Specialist Nurse- Led Clinic | 🞏 | 🞏 | 🞏 | 🞏 | 🞏 |
| **6** | Specialist Pharmacist- Led Clinic | 🞏 | 🞏 | 🞏 | 🞏 | 🞏 |
| **95** | Other | 🞏 | 🞏 | 🞏 | 🞏 | 🞏 |

**D5**

How often do you see the following types of patients in clinic?

*Please select one per patient group.*

|  |  | **Optimally responding CP-CML** | **Warning CML patient** | **Failure CP-CML** | **Intolerant patient** | **TFR patient** |
| --- | --- | --- | --- | --- | --- | --- |
| **1** | Monthly | 🞏 | 🞏 | 🞏 | 🞏 | 🞏 |
| **2** | Every 2 months | 🞏 | 🞏 | 🞏 | 🞏 | 🞏 |
| **3** | Every 3 months | 🞏 | 🞏 | 🞏 | 🞏 | 🞏 |
| **4** | Every 6 months | 🞏 | 🞏 | 🞏 | 🞏 | 🞏 |
| **5** | Once a year | 🞏 | 🞏 | 🞏 | 🞏 | 🞏 |
| **95** | Other | 🞏 | 🞏 | 🞏 | 🞏 | 🞏 |

**D6**

What is the most common way you review your patients in clinic?

*Please select one per patient type.*

|  |  | **Optimally responding CP-CML** | **Warning CML patient** | **Failure CP-CML** | **Intolerant patient** | **TFR patient** |
| --- | --- | --- | --- | --- | --- | --- |
| **1** | In-person | 🞏 | 🞏 | 🞏 | 🞏 | 🞏 |
| **2** | Telephone | 🞏 | 🞏 | 🞏 | 🞏 | 🞏 |
| **3** | Videocall | 🞏 | 🞏 | 🞏 | 🞏 | 🞏 |
| **4** | Hybrid | 🞏 | 🞏 | 🞏 | 🞏 | 🞏 |

**D7**

If you monitor your patients remotely, how do you obtain the patient’s blood samples?

| **1** | GP |
| --- | --- |
| **2** | Phlebotomy hub |
| **3** | Hospital setting |
| **95** | Other |
| **4** | I don’t monitor any of my patients remotely |

**D8**

Do you use any of the following tools or resources to monitor patients in CML?~~?~~

*Please select all that apply*

| **1** | Patient Diary |
| --- | --- |
| **2** | Patient App |
| **3** | MDASI-CML |
| **97** | Other QOL measuring tool (please specify) |
| **4** | CML CNS Assessment |
| **95** | Other |
| **96** | I don’t have access nor use any of the above |

**D9**

Which additional tools or support do you feel would improve the monitoring and management of CML patients?

**Section 5: Patient Management**

**E1**

How do you define resistance to TKI therapy in your routine practice?

*Select all that apply.*

| **1** | As per ELN2013 guidelines |
| --- | --- |
| **2** | As per ELN2020 guidelines |
| **3** | As per BSH 2020 Guidelines |
| **4** | Not achieving complete hematologic response (CHR) by 3 months |
| **5** | No CCyR or BCR::ABL1 IS <1% by 6 months |
| **6** | No CCyR or BCR::ABL1 IS <1% by 12 months |
| **7** | No MMR by 12 months |
| **8** | Presence of mutation |
| **9** | Any loss of prior response (MMR, CCyR, CHR) |
| **10** | CCyR or BCR::ABL1 IS >1% |
| **95** | Other (please specify) |

**E2**

What actions do you currently take in the following situations?

*Select all that apply for each.*

|  |  | **A warning ELN response** | **When a patient has had more than one warning ELN response** | | **A failure ELN response** | |
| --- | --- | --- | --- | --- | --- | --- |
| **96** | No action | 🞏 | | 🞏 | | 🞏 |
| **1** | Evaluate compliance | 🞏 | | 🞏 | | 🞏 |
| **2** | More frequent monitoring | 🞏 | | 🞏 | | 🞏 |
| **3** | Switch therapy | 🞏 | | 🞏 | | 🞏 |
| **4** | Dose modification | 🞏 | | 🞏 | | 🞏 |
| **5** | BCR::ABL1 tyrosine kinase domain mutation analysis | 🞏 | | 🞏 | | 🞏 |

**E3**

Would there be a reason you would not perform BCR::ABL1 tyrosine kinase domain mutational analysis in TKI resistant patients?

*Select all that apply*

| **1** | Turnaround time too slow |
| --- | --- |
| **2** | Does not impact treatment decision |
| **3** | Too early to change treatment |
| **4** | Not available routinely |
| **5** | Cost |
| **96** | Not applicable |

**E4**

Do you wait for BCR::ABL1 tyrosine kinase domain mutation analysis results prior to switching patients to an alternative TKI?

| **1** | Yes |
| --- | --- |
| **2** | No |
| **3** | Sometimes |

**E5**

Do you have a reason for not waiting for the result?

**E6**

Which of the following would you define as intolerance to a TKI therapy?

*Select all that apply.*

| **1** | CTCAE grade 1 |
| --- | --- |
| **2** | CTCAE persistent grade 1 |
| **3** | CTCAE grade 2 |
| **4** | CTCAE persistent grade 2 |
| **5** | CTCAE grade ≥ 3 |
| **6** | Impact on quality of life (QOL)/Patient reported issues |
| **7** | Tolerability affecting compliance |
| **95** | Other (please specify) |

**E7**

Which of the following actions do you consider if a patient is intolerant to TKI therapy?

|  |  | **Intolerant to 1st Line TKI** | **Intolerant to 2nd Line TKI** | | **Intolerant to 3rd Line TKI** | |
| --- | --- | --- | --- | --- | --- | --- |
| **1** | No Action | 🞏 | | 🞏 | | 🞏 |
| **2** | Evaluate Compliance | 🞏 | | 🞏 | | 🞏 |
| **3** | More frequent follow up | 🞏 | | 🞏 | | 🞏 |
| **4** | Dose interruption | 🞏 | | 🞏 | | 🞏 |
| **5** | Dose reduction | 🞏 | | 🞏 | | 🞏 |
| **6** | Introduction or increase of concomitant medication | 🞏 | | 🞏 | | 🞏 |
| **7** | Consideration of Drug Interactions | 🞏 | | 🞏 | | 🞏 |
| **8** | Conduct further investigations | 🞏 | | 🞏 | | 🞏 |
| **9** | Refer to another specialty (e.g. cardiologist) | 🞏 | | 🞏 | | 🞏 |
| **10** | Switch Therapy | 🞏 | | 🞏 | | 🞏 |
| **11** | Refer to CML specialist centre | 🞏 | | 🞏 | | 🞏 |
| **95** | Other | 🞏 | | 🞏 | | 🞏 |

**E8**

Which of the following actions do you consider if a patient is intolerant to TKI therapy?

|  |  | **Grade 1 or 2 CTCAE** | **Persistent Grade 1 or 2 CTCAE** | | **Grade ≥3 CTCAE** | |
| --- | --- | --- | --- | --- | --- | --- |
| **1** | No Action | 🞏 | | 🞏 | | 🞏 |
| **2** | Evaluate Compliance | 🞏 | | 🞏 | | 🞏 |
| **3** | More frequent follow up | 🞏 | | 🞏 | | 🞏 |
| **4** | Dose interruption | 🞏 | | 🞏 | | 🞏 |
| **5** | Dose reduction | 🞏 | | 🞏 | | 🞏 |
| **6** | Introduction or increase of concomitant medication | 🞏 | | 🞏 | | 🞏 |
| **7** | Consideration of Drug Interactions | 🞏 | | 🞏 | | 🞏 |
| **8** | Conduct further investigations | 🞏 | | 🞏 | | 🞏 |
| **9** | Refer to another specialty (e.g. cardiologist) | 🞏 | | 🞏 | | 🞏 |
| **10** | Switch Therapy | 🞏 | | 🞏 | | 🞏 |
| **11** | Refer to CML specialist centre | 🞏 | | 🞏 | | 🞏 |
| **95** | Other | 🞏 | | 🞏 | | 🞏 |

**E9**

Please estimate what percentage of your CML-CP patients would have changed their treatment in the first two years since diagnosis

|  |  | **% of patients** |
| --- | --- | --- |
| **1** | At least once |  |
| **2** | More than once |  |

**Section 6: Treatment Free Remission (TFR)**

**F1**

When do you most commonly consider TFR for a patient?

*Please select one.*

| **1** | At Diagnosis |
| --- | --- |
| **2** | After Patient Request |
| **3** | Achievement of DMR |
| **4** | Maintenance of DMR > 2 years |
| **95** | Other (please specify) |
| **96** | Never |

**F2**

What guidelines do you follow in the management of TFR?

*Select all that apply.*

| **1** | Local |
| --- | --- |
| **2** | ELN |
| **3** | BSH |
| **4** | ESMO |
| **5** | NCCN |
| **6** | As per DESTINY protocol |
| **95** | Other |

**F3**

For those patients who manage to attempt a trial of TFR, what is your observed success rate of TFR maintenance at 12 months?

*Please select one.*

| **1** | 0-20% |
| --- | --- |
| **2** | 21-40% |
| **3** | 41-60% |
| **4** | 61-80% |
| **5** | 81-100% |
| **6** | Never attempted TFR |
| **95** | Unsure |

**F4**

What proportion of your patients have suffered from TKI-withdrawal syndrome?

*Please select one.*

| **1** | 0-20% |
| --- | --- |
| **2** | 21-40% |
| **3** | 41-60% |
| **4** | 61-80% |
| **5** | 81-100% |
| **95** | Unsure |

**F5**

Which factors influence your consideration for treatment free response?

|  |  | | **Never** | | **Sometimes** | **Always** | |
| --- | --- | --- | --- | --- | --- | --- | --- |
| **1** | Patient age | 🞏 | | 🞏 | | 🞏 |  |
| **2** | Low/Intermediate ELTS Score | 🞏 | | 🞏 | | 🞏 |  |
| **3** | High ELTS Score | 🞏 | | 🞏 | | 🞏 |  |
| **4** | High-risk features | 🞏 | | 🞏 | | 🞏 |  |
| **5** | Typical e13a2 or e14a2 *BCR::ABL1* Transcript | 🞏 | | 🞏 | | 🞏 |  |
| **6** | Atypical *BCR::ABL1* Transcript | 🞏 | | 🞏 | | 🞏 |  |
| **7** | Motivated patient | 🞏 | | 🞏 | | 🞏 |  |
| **8** | Patient on approved TKI therapy >3 years | 🞏 | | 🞏 | | 🞏 |  |
| **9** | Patient on approved TKI therapy >5 years | 🞏 | | 🞏 | | 🞏 |  |
| **10** | Duration of DMR > 2 years | 🞏 | | 🞏 | | 🞏 |  |
| **11** | Duration of MR4.0 > 3 years | 🞏 | | 🞏 | | 🞏 |  |
| **12** | Duration of MR4.5 > 2 years | 🞏 | | 🞏 | | 🞏 |  |
| **13** | Patient on 1st line TKI | 🞏 | | 🞏 | | 🞏 |  |
| **14** | Patient on 2nd Line TKI due to prior intolerance | 🞏 | | 🞏 | | 🞏 |  |
| **15** | No prior treatment failure | 🞏 | | 🞏 | | 🞏 |  |
| **16** | Patient Pregnancy Planning | 🞏 | | 🞏 | | 🞏 |  |
| **17** | Patient with impaired QOL on TKI | 🞏 | | 🞏 | | 🞏 |  |
| **18** | CV risk factors | 🞏 | | 🞏 | | 🞏 |  |
| **19** | Prior BCR::ABL1 tyrosine kinase domain mutation | 🞏 | | 🞏 | | 🞏 |  |
| **95** | Other | 🞏 | | 🞏 | | 🞏 |  |

**F6**

Do you discuss potential TFR patients in an MDT prior to attempting treatment-free remission?

*Please select one.*

| **1** | Yes, all patients |
| --- | --- |
| **2** | Almost all patients |
| **3** | Most patients |
| **4** | Some patients |
| **5** | Never |

**F7**

For patients in TFR, aside from molecular monitoring do you perform any additional tests?

*Select all that apply.*

| **1** | Full Blood Count |
| --- | --- |
| **2** | Biochemical Tests |
| **95** | Other tests (please specify) |
| **3** | None of the above |

**Section 7: Unmet Need**

**G1**

What do you believe is the current medical unmet need in CML in each of the following settings?

*Select all that apply at each line.*

|  |  | **1^st^ line setting** | **2^nd^ line setting** | | **3^rd^ line setting** | |
| --- | --- | --- | --- | --- | --- | --- |
| **1** | Intolerance to current options | 🞏 | | 🞏 | | 🞏 |
| **2** | Resistance to current options | 🞏 | | 🞏 | | 🞏 |
| **3** | Lack of treatment options | 🞏 | | 🞏 | | 🞏 |
| **4** | TKI toxicity | 🞏 | | 🞏 | | 🞏 |
| **5** | Improving QOL | 🞏 | | 🞏 | | 🞏 |
| **6** | T315I | 🞏 | | 🞏 | | 🞏 |
| **7** | Achieving TFR | 🞏 | | 🞏 | | 🞏 |
| **8** | Maintaining TFR | 🞏 | | 🞏 | | 🞏 |
| **9** | Fitness for transplant | 🞏 | | 🞏 | | 🞏 |
| **95** | Other (please specify) | 🞏 | | 🞏 | | 🞏 |

**G2**

Do you think that your local clinical team could benefit from further education in any of the following areas of managing CML?

*Select all that apply*

| **1** | ELN 2020 guidelines |
| --- | --- |
| **2** | BSH 2020 Guidelines |
| **3** | Molecule testing techniques & report interpretation |
| **4** | Management of TFR |
| **5** | AE management |
| **6** | Comorbidity management |
| **7** | Cardiovascular risk management |
| **96** | No education needed |
| **95** | Other (please specify) |

**G3**

Who in your department would benefit most from this training?

*Select all that apply*

| **1** | Yourself |
| --- | --- |
| **2** | Other consultants |
| **3** | Clinical nurse specialists (CNS) |
| **4** | Specialist Trainees |
| **5** | Associate Specialists |
| **6** | Staff Grades |
| **7** | Physician Associates |
| **95** | Other |
