# Supplementary material for: Adapting UK Clinical Practise in Chronic Myeloid Leukaemia in Chronic Phase to Contemporary Management Recommendations: The ADAPT 2.0 Survey
Source: Eur J Haematol. 2026 Jun 25;117(4):1072–80. doi: 10.1111/ejh.70226 (PMC13542934; doi:10.1111/ejh.70226)
Supplement: Supplementary file 2 [file EJH-117-1072-s002.docx]

**Supplemental Materials**

**Adapting UK clinical practise in chronic myeloid leukaemia in chronic phase to contemporary management recommendations: the ADAPT 2.0 survey**

Dragana Milojkovic^1,*^, Nicholas C. P. Cross^2^, Ketul Desai^3^, Kathryn Frewin^3^, Mhairi Copland^4^

^1^ Centre for Haematology, Faculty of Medicine, Imperial College London, and Department of Haematology, Hammersmith Hospital, Imperial College Healthcare NHS Trust, London, UK

^2^ Faculty of Medicine, University of Southampton, Southampton, UK

^3^ Novartis Pharmaceuticals Ltd, London, UK

^4^ Paul O'Gorman Leukaemia Research Centre, School of Cancer Sciences, College of Medical, Veterinary and Life Sciences, University of Glasgow, Glasgow, UK.

**Index of Supplemental Tables and Figures**

|  | **Page** |
| --- | --- |
| **Supplementary Table 1.** Factors affecting TKI selection for patients with CML-CP | **3** |
| **Supplemental Figure 1.** Information routinely captured in patient’s notes regarding CV risk | **4** |
| **Supplemental Figure 2.** CV assessment tools used to assess patients with CML prior to first TKI | **5** |
| **Supplemental Figure 3.** Risk scoring tools used to assess patients with CML-CP prior to first TKI | **5** |
| **Supplemental Figure 4.** Frequency of considering 2G TKI as 1L treatment for CML-CP | **6** |
| **Supplemental Figure 5.** Additional tests performed for routine follow-up | **7** |
| **Supplemental Figure 6.** Ways to review patients in the clinic according to interface use and pathways to obtain blood samples | **8** |
| **Supplemental Figure 7.** Tools and resources used to monitor patients with CML and additional tools required by HCPs | **9** |
| **Supplemental Figure 8.** Reasons for not performing *BCR::ABL1* tyrosine kinase domain mutational analysis in TKI-resistant patients | **10** |
| **Supplemental Figure 9.** Actions considered if patient is intolerant to TKI therapy by therapy line and AE severity | **11** |
| **Supplemental Figure 10.** Less important factors affecting consideration for TFR | **12** |
| **Supplemental Figure 11.** Educational requirements of clinical teams managing CML and HCPs who would benefit from further education | **13** |

**Supplemental Tables**

**Supplemental Table 1.** Factors affecting TKI selection for patients with CML-CP

| **Category, %** | **1L** | **2L after 1L intolerance** | **2L after 1L resistance** | **3L after 2L intolerance** | **3L after 2L resistance** |
| --- | --- | --- | --- | --- | --- |
| Guidelines | 97 | 87 | 92 | 85 | 87 |
| Comorbidities | 90 | 100 | 100 | 100 | 92 |
| Aiming for TFR | 82 | 62 | 31 | 33 | 15 |
| Safety profile | 79 | 92 | 90 | 90 | 82 |
| CV risk score | 79 | 90 | 90 | 87 | 82 |
| Efficacy | 77 | 79 | 79 | 79 | 82 |
| QoL | 74 | 85 | 74 | 85 | 77 |
| Tolerability | 72 | 90 | 77 | 90 | 74 |
| Patient choice | 72 | 82 | 67 | 79 | 67 |
| Age | 72 | 62 | 59 | 51 | 54 |
| CML progression risk score | 69 | 38 | 38 | 31 | 36 |
| Dosing regimen | 51 | 51 | 38 | 41 | 31 |
| Physician experience | 46 | 51 | 49 | 54 | 51 |
| Standard local management choice | 28 | 23 | 21 | 21 | 21 |
| Cost | 23 | 3 | 3 | 3 | 3 |
| Other | 5 | 3 | 5 | 5 | 8 |

% of HCPs, top 3 highlighted for each patient type. Results were not mutually exclusive.

1L, first line; 2L, second line; 3L, third line; CML, chronic myeloid leukaemia; CV, cardiovascular; QoL, quality of life; TFR, treatment-free remission.

**Supplemental Figures**

**Supplemental Figure 1.** Information routinely captured in patient’s notes regarding CV risk. BMI, body mass index; CV, cardiovascular; ECG, electrocardiogram; ECHO, echocardiogram; HCP, healthcare professional; LDL, low-density lipoprotein.

**Supplemental Figure 2.** CV assessment tools used to assess patients with CML prior to first TKI. CML, chronic myeloid leukaemia; CV, cardiovascular; GP, General Practitioner; HCP, healthcare professional; TKI, tyrosine kinase inhibitor.

**Supplemental Figure 3**. Risk scoring tools used to assess patients with CML-CP prior to first TKI. CML-CP, chronic myeloid leukaemia in chronic phase; ELTS, EUTOS long-term survival; HCP, healthcare professional; TKI, tyrosine kinase inhibitor.

**Supplemental Figure 4**. Frequency of considering 2G TKI as 1L treatment for CML-CP. Younger patients were defined as <40 years old and older patients as >60 years old. 1L, first line; 2G, second generation; CML-CP, chronic myeloid leukaemia in chronic phase; CV, cardiovascular; ELTS, EUTOS long-term survival; HCP, healthcare professional; TFR, treatment-free remission; TKI, tyrosine kinase inhibitor.

**Supplemental Figure 5**. Additional tests performed for routine follow-up. CBA, chromosome banding analysis; FISH, fluorescence in situ hybridisation; HCP, healthcare professional; NGS, next generation sequencing.

**Supplemental Figure 6.** Ways to review patients in the clinic according to interface use (**A**) and pathways to obtain blood samples (**B**). CML, chronic myeloid leukaemia; CML-CP, chronic myeloid leukaemia in chronic phase; GP, General Practitioner; HCP, healthcare professional; TFR, treatment-free remission.

**A**

**B**

**Supplemental Figure 7.** Tools and resources used to monitor patients with CML (**A**) and additional tools required by HCPs (**B**). CML, chronic myeloid leukaemia; CNS, central nervous system; GP, General Practitioner; HCP, healthcare professional; MDASI, MD Anderson symptom inventory; TFR, treatment-free remission.

**A**

**B**

**Supplemental Figure 8.** Reasons for not performing *BCR::ABL1* tyrosine kinase domain mutational analysis in TKI-resistant patients. CML, chronic myeloid leukaemia; HCP, healthcare professional; MDASI, MD Anderson symptom inventory; TFR, treatment-free remission; TKI, tyrosine kinase inhibitor.

**Supplemental Figure 9.** Actions considered if patient is intolerant to TKI therapy by therapy line (**A**) and AE severity (**B**). 1L, first line; 2L, second line; 3L, third line; AE, adverse event; CML, chronic myeloid leukaemia; CTCAE, Common Terminology Criteria for Adverse Events; HCP, healthcare professional; TKI, tyrosine kinase inhibitor.

**A**

**B**

**Supplemental Figure 10.** Less important factors affecting consideration for TFR. 2L, second line; CV, cardiovascular; ELTS, EUTOS long-term survival score; HCP, healthcare professional; TFR, treatment-free remission; TKI, tyrosine kinase inhibitor.

**Supplemental Figure 11.** Educational requirements of clinical teams managing CML (**A**) and HCPs who would benefit from further education (**B**). AE, adverse event; BSH, British Society for Haematology; CML, chronic myeloid leukaemia; ELN, European LeukemiaNet; HCP, healthcare professional; TFR, treatment-free remission.

**B**

**A**
